# The Role of the Gut Microbiota in the Pathogenesis of Diabetes

**DOI:** 10.3390/ijms23010480

**Published:** 2022-01-01

**Authors:** Weronika Bielka, Agnieszka Przezak, Andrzej Pawlik

**Affiliations:** Department of Physiology, Pomeranian Medical University in Szczecin, 70-111 Szczecin, Poland; weronika.bielka@wp.pl (W.B.); agn-prze@wp.pl (A.P.)

**Keywords:** diabetes, microbiota, therapy

## Abstract

Diabetes mellitus is a significant clinical and therapeutic problem because it can lead to serious long-term complications. Its pathogenesis is not fully understood, but there are indications that dysbiosis can play a role in the development of diabetes, or that it appears during the course of the disease. Changes in microbiota composition are observed in both type 1 diabetes (T1D) and type 2 diabetes (T2D) patients. These modifications are associated with pro-inflammation, increased intestinal permeability, endotoxemia, impaired β-cell function and development of insulin resistance. This review summarizes the role of the gut microbiota in healthy individuals and the changes in bacterial composition that can be associated with T1D or T2D. It also presents new developments in diabetes therapy based on influencing the gut microbiota as a promising method to alter the course of diabetes. Moreover, it highlights the lacking data and suggests future directions needed to prove the causal relationship between dysbiosis and diabetes, both T1D and T2D.

## 1. Introduction

Diabetes is a group of metabolic diseases characterized by hyperglycemia caused by the direct or indirect deficiency of insulin. Type 1 diabetes (T1D) is an autoimmune disease in which antibodies are produced against various elements of pancreatic β-cells; the islets producing insulin become deteriorated and, eventually, are completely destroyed, which causes a lack of insulin [1]. Type 2 diabetes (T2D) is triggered by insulin resistance (IR), which leads to an increased demand of peripheral tissues for insulin and, as a consequence, causes the functional failure of β-cells [2]. Inadequate metabolic management of diabetes can lead to serious long-term complications, including retinopathy, chronic kidney disease, neuropathy and cardiovascular disease, and increased mortality [3,4,5]. In 2019, the International Diabetes Federation reported that the number of people with diabetes was estimated to be 463 million and would increase to 700 million by 2045 [6]. This great number of people who are at the risk of diabetes, indicates the need to search for further explanations for diabetes pathogenesis, which, as a consequence, can lead to the development of new strategies aimed at preventing the disease or alleviating its course.

The gut microbiota contribute to the proper functioning of human organisms [7]. They create a dynamic ecosystem that is modulated by internal and external factors. Altered composition of intestinal bacteria can participate in the pathogenesis of disorders, such as obesity, diabetes and heart failure [8,9,10], the prevalence of which is still increasing in the world. Thus, it is of great importance to discover whether the bacteria contribute to the development of these diseases of civilization. It can not only help to modify their course or to delay the appearance of complications, but also to prevent the onset of the disorders. In this review, we describe the differences in the gut microbiota in patients with T1D and T2D in comparison to healthy individuals, explain the probable impact of altered bacterial composition on the host organism and indicate potential therapeutic targets aimed at the microbiota, which can influence the course of diabetes. Moreover, we focus on lacking data in the area concerning the gut microbiota, point out the weak points of studies conducted so far and suggest future directions needed to prove the causal relationship between dysbiosis and diabetes, both T1D and T2D.

## 2. The Gut Microbiota—Role in Host Homeostasis

The term “the gut microbiota” refers to more than 10^14^ bacteria that settle in the gastrointestinal tract, in which they perform a variety of functions, not all yet fully understood. The “microbiome” is considered as the genome of the whole microbiota. The number of bacterial cells was estimated to be about 10 times higher than the number of human cells [11], but nowadays it is known that the numbers of bacterial and human cells are similar [12]. The microbiota of healthy adults consist of six phyla—*Firmicutes* and *Bacteroidetes*, which are the major groups of bacteria, but also *Proteobacteria*, *Actinobacteria*, *Fusobacteria* and *Verrucomicrobia*. The microbiota of adults include a wide range of species (about 500–1000) belonging to two main phyla—*Firmicutes* and *Bacteroidetes*—which makes the bacterial community in every individual specific and unique [13,14]. Maintaining this diversity and tight homeostasis between bacteria is supposed to be essential to keep human health in good condition, and dysbiosis can contribute to the development of metabolic diseases, such as obesity or diabetes [8,15,16].

The intestinal microbiota contribute to carbohydrate metabolism by hydrolyzing and fermenting polysaccharides delivered with food. As a consequence, monosaccharides and short-chain fatty acids (SCFAs) are produced, which can influence the colon locally, but can also be absorbed by the host into the circulation and influence the metabolism of different organs [17]. SCFAs, consisting predominantly of acetate, propionate and butyrate, act through G protein-coupled receptors—GPR41, GPR43, and GPR109A—and histone deacetylase [18,19,20,21,22]. These receptors are expressed in various tissues; for instance, adipose tissue, distal ileum, colon, lymph nodes and immune cells—neutrophiles and monocytes [19,20,21]. *Bacteroidetes* mainly generate acetate and propionate, and *Firmicutes* produce butyrate [23]. Species of the genus *Bifidobacterium*, belonging to *Actinobacteria*, directly produce a great amount of the SCFA acetate, but they also produce lactate, which is metabolized by other bacteria to butyrate [24,25]. SCFAs regulate the proper function, motility and integrity of the gastrointestinal tract. They probably improve glucose homeostasis and strengthen satiety by increasing the production of the glucagon-like peptide-1 (GLP-1) in the intestine [26]. They influence insulin sensitivity and glucose tolerance by mediating the glycemic response, help to maintain the integrity of the gut epithelium via inducing mucin synthesis and improve the intestinal barrier by facilitating tight junction assembly [27,28]. They can also lead to the increased secretion of peptide YY and leptin, affecting satiety [29]. Moreover, SCFAs support the host immune system, influencing the functions of macrophages, dendritic cells, T cells and B cells, and, as a consequence, prevent the invasion of pathogens, such as *Shigella* and entero-hemorrhagic *Escherichia coli* [24,30,31,32]. The gut microbiota are crucial for proper intestinal barrier functioning. They provide adequate energy for the proliferation of epithelial cells, as butyrate is the main energy source for colonocytes, as well as stimulating the immune system to properly respond to pathogens [33,34,35]. *Actinobacteria* contribute to the maintenance of intestinal barrier homeostasis [36].

The gut microbiota are able to synthesize branched-chain amino acids (BCAAs)—leucine, isoleucine and valine [37]. These molecules can be considered as indicators of IR and predictors of diabetes mellitus development, because the serum metabolomes of patients suffering from IR or T2D contain an increased amount of BCAAs [38,39]. It has been proved that the main species positively associated with IR are *Prevotella copri* and *Bacteroides vulgatus*. In turn, the leading species negatively associated with IR are *Butyrivibrio crossotus* and *Eubacterium siraeum* [38]. The increased intake of BCAAs in food is associated with a higher risk of IR appearance, and decreased consumption can cause an improvement of postprandial insulin sensitivity [40,41].

The primary bile acids (Bas) are synthesized from cholesterol in the liver and secreted with bile into the gut lumen. Further, they are metabolized by the microbiota to secondary Bas and mostly reabsorbed into circulation, influencing different host processes [42]. Their main role is the participation in the process of digestion and absorption of lipids and vitamins soluble in lipids. They contribute to the proper regulation of carbohydrate and lipid metabolism, as well as to the regulation of energy expenditure via the farnesoid X receptor (FXR) and TGR5 [42,43]. Depending on the localization of activation of FXR (in the intestine or in the liver), they can show features that are either protective from or conductive to steatosis and obesity [44,45,46]. Moreover, these receptors play a role in the production and release of GLP-1 by L cells [47,48]. The microbiota not only produce secondary BAs, but also regulate their uptake and participate in the synthesis of primary BAs by regulating the crucial enzymes [49].

The gut microbiota play a crucial role in preventing infectious diseases by occupying host niches, which can make the host resistant to colonization by pathogens [50].

The gut microbiota also participate in the proper functions of the immune system, which is a complicated mechanism consisting of a variety of different actions. For instance, they take part in the process of inflammatory cytokine production or stimulate the proliferation of group 3 innate lymphoid cells in the colon [51,52]. Moreover, they affect CD8+ T cell memory and macrophages through one of the SCFAs, butyrate [53,54]. The gut microbiota also synthesize and metabolize vitamin K and B group vitamins, as well as metabolizing drugs and toxins [55].

**Figure 1:**
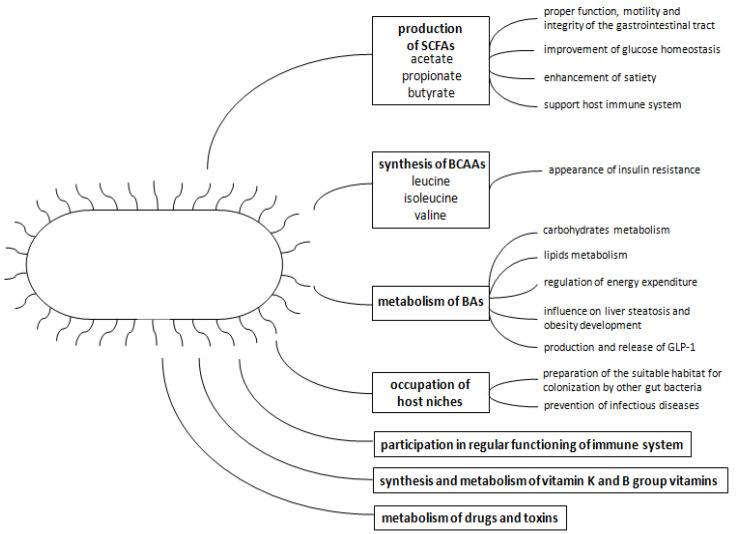
The role of the gut microbiota in maintaining host homeostasis.

Bacteria belonging to the phyla *Proteobacteria* and *Actinobacteria* are less abundant than *Firmicutes* and *Bacteroidetes* [56]. A temporary dominance of *Proteobacteria*, especially *Enterobacteriaceae*, has been found in newborn mice [57]. By consuming oxygen and altering the pH, *Proteobacteria* species, facultative anaerobes, play a key role in preparing the suitable habitat of the infant gut for colonization by strict anaerobes [58]. Vaughn et al. have shown that mice fed with a high-fat diet are characterized by microbiota changes, especially an increase in the level of *Proteobacteria*, which are probably directly associated with the reorganization of vagal afferents and microglia activation in the nucleus of the solitary tract [59]. A short summary of the role of the gut microbiota is presented in Figure 1.

## 3. Changes in the Composition of the Gut Microbiota in Patients with T1D

The stability, connectivity, abundance and composition of the intestinal microbiota are probably associated with the development of T1D [60]. Several studies have provided information about an altered gut microbiota in T1D-affected patients. Giongo et al. indicated that a high *Firmicutes*/*Bacteroidetes* ratio and the instability of the microbiota can be one of the early diagnostic markers of developing autoimmune disorders, such as T1D [61]. De Goffau et al. examined the composition of the gut microbiota at the onset of T1D in young children. In diabetic children, there was an increased level of *Bacteroidetes* and *Streptococcus mitis*, while in healthy controls there was a higher prevalence of the butyrate producers *Lactobacillus plantarum* and *Clostridium* clusters *IV* and *XIVa* [62]. Similar conclusions were drawn by Mejía-León et al. At diagnosis, T1D-affected patients had a dominance of *Bacteroides* and controls had a higher level of *Prevotella*, but after 2 years of treatment with insulin, the gut microbiota of patients and of controls were similar [63]. The fecal microbiota in early-onset T1D were also analyzed by metagenomic sequencing in the TEDDY longitudinal study. Vatanen et al. indicated that in stool samples from children diagnosed with T1D, the levels of *Roseburia hominis*, *Alistipes shahii* and *Bifidobacterium pseudocatenulatum* were higher, whereas in controls without T1D, levels of *Lactococcus lactis* and *Streptococcus thermophilus* were raised. The control group of children not only had more species common in dairy products, but also their microbiota consisted of more genera associated with the biosynthesis of SCFAs and fermentation. This finding supports the theory of the protective effects of SCFAs in T1D [64]. Pellegrini et al. showed a characteristic inflammatory profile and microbiota in the duodenal mucosa of patients with T1D. In the samples of mucosa, increased inflammation and monocyte/macrophage lineage infiltration were observed. In patients with T1D, the level of *Firmicutes* and the *Firmicutes*/*Bacteroidetes* ratio were raised and the levels of *Proteobacteria* and *Bacteroidetes* were reduced [65]. Moreover, in the study of Siljander et al., the pro-inflammatory environment in the gut in children developing T1D was related to a decreased level of *Firmicutes* and increased amount of *Bacteroidetes* [66]. Dissimilar conclusions between studies can result from the distinct methods used in the research, but also from the differences in the gut microbiota between individuals as a consequence of their geographical location [67]. A short comparison of these changes in the gut microbiota in individuals with diabetes is presented in Table 1.

## 4. The Potential Role of the Gut Microbiota in the Development of T1D

T1D is defined as a β-cell-mediated pro-inflammatory state, induced by both innate and adaptive immunity [75]. Specific human leucocyte antigen (HLA) genotypes, such as DQ2, DQ8, DR3 and some DR4 alleles, are the main factors of a genetic predisposition to T1D development [76]. However, in family and twin studies, it has been proved that only 20–30% of genetically predisposed individuals carrying these alleles will develop T1D [77]. In disease onset, equally important is the impact of environmental factors, such as the way of feeding, diet or exposure to viruses in early childhood [78]. Furthermore, altered gut bacterial composition can be associated with the pathogenesis of insulin dysfunction and T1D [53]. Nevertheless, most studies investigating the concept that gut microbiota affect the pathogenesis of T1D are performed on mouse models, while there are still an insufficient number of human studies to prove it.

During early childhood, such processes occur as the development of the immune system, maturation of the gut microbiota and appearance of the first autoantibodies bound to T1D [65]. Among the factors that can modify the composition of the intestinal microbiota are breastfeeding, nutrition, route of delivery, use of antibiotics and exposure to the microbes in the environment [79,80]. Their action can result in intestinal barrier disruption and defective maturation of the immune response, eventually leading to T1D progression later in life [81]. Moreover, the genetic set-up of the host can interact with the intestinal microbiota, causing changes in the microbial composition, activation of immunity and susceptibility to T1D [82,83].

Dysbiosis, defined as the repetitive or prolonged deviation from optimal microbial homeostasis, can cause the loss of self-tolerance and the spread of effector cells and pro-inflammatory signals in the organism [65]. These processes coincide with the increased permeability of the intestinal wall, translocation of microbial material through the epithelium and enhanced presentation of antigens, as well as autoantigens [65]. This leads to the activation of the pro-inflammatory pathway in the intestine, lymph nodes and pancreas [65]. Furthermore, the exocrine function of the pancreas, quality of the mucosal barrier and adhesion of the microvilli are depleted in patients with T1D [84,85]. Some members of *Bifidobacterium*, *Bacteroides* and *Ruminococcus* can cause mucin degradation and impair the integrity of the mucosal barrier [86]. Intestinal inflammation and the reduction of SCFAs caused by dysbiosis can be crucial to the pathogenesis of T1D [87]. The clinical onset of T1D is probably preceded by heightened gut permeability [88]. An adequate amount of butyrate, produced mainly by *Firmicutes*, leads to appropriate mucin synthesis and enhances tight junctions in the intestine [89,90]. Butyrate also shows anti-inflammatory properties and decreases bacterial transport through the epithelial cells [91]. A butyrate diet helped to increase the amount and function of regulatory T cells [92], whereas acetate- and butyrate-yielding diets decreased serum concentrations of diabetogenic cytokines, such as IL-21, and enhanced gut integrity. This type of diet allowed a reduction in the incidence of diabetes in NOD (non-obese diabetic) mice. Moreover, female NOD mice had a larger number of pancreatic islets with no infiltration [93].

**Figure 2:**
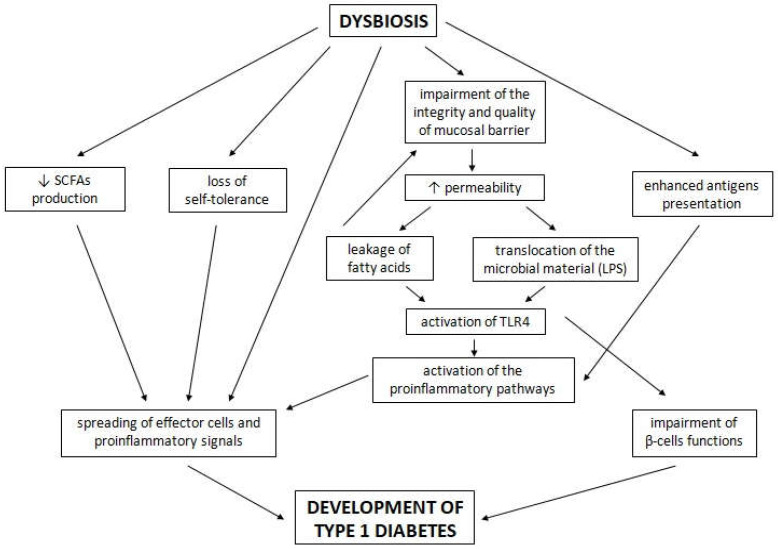
The possible influence of dysbiosis on type 1 diabetes development. A description is given in the text above.

The alteration of intestinal microbes can induce the leakage of fatty acids and lipopolysaccharides (LPSs) by destroying the intestinal mucosal barrier. This causes the activation of toll-like receptor 4 (TLR4), which results in metabolic inflammation [94]. TLRs are engaged in maturation of dendritic cells and recognizing pathogen-associated molecular patterns derived from microbiota [95]. They contribute to protecting the host from infectious microbes. MyD88 is an adaptor not only for TLRs and interleukin 1, but also other innate immune receptors. A defect of MyD88 can alter the composition of microflora in the distal part of the intestine [92]. Moreover, in NOD mice, the knockout of MyD88 protected against T1D development [93]. LPS is a bacterial endotoxin and one of the components of the outer membrane of Gram-negative bacterial species, and probably acts as a molecular link between gut microbiota, inflammation and T1D [53]. In a case-control study, it was proved that patients with T1D have higher circulating LPS levels than those without diabetes [96]. LPSs can be involved in diabetes development, because they lead to the impairment of pancreatic β-cell function and increase the level of pro-inflammatory cytokines [97]. In mouse models, an oral injection of *E. coli* LPS improved local immunity, while an intraperitoneal injection of *E. coli* LPS improved the autoimmune response and decreased the incidence of T1D [98,99]. However, it is difficult to conclude whether microbial alteration is causal or consequential for T1D development. The possible influence of dysbiosis on T1D development is presented in Figure 2. Most current studies mainly show the involvement of intestinal microbiota in the β-cell autoimmunity process, and do not focus on an explanation of whether gut microbiota activate T1D. On the one hand, the state of dysbiosis during the maturation of the immune system can destroy self-tolerance and control of the inflammatory response, which can eventually lead to increased susceptibility to immune-mediated diseases, such as T1D. However, on the other hand, pro-inflammatory intestinal dysbiosis and changed microbial diversity can cause T1D activation after seroconversion. How the mechanisms of relatively local intestinal inflammation spread to an autoimmune process of the whole organism is not clear enough [66]. It is necessary to perform new interventional studies and not only observational ones. There exists a strong need to prove the causal relationship between T1D and intestinal microbiota, and the exact mechanisms that participate in the processes described above.

## 5. Changes in the Composition of the Gut Microbiota in Patients with T2D

Similarly to T1D, the microbiota in patients with T2D differ from those occurring in healthy individuals [16]. The main change seen in various research is an increase in the amount of opportunistic pathogens and a decrease in bacteria producing butyrate, one of the SCFAs [97]. One of the first studies on the microbiota of subjects with T2D, conducted by Larsen et al., showed decreased levels of the phylum *Firmicutes* and class *Clostridia*. Moreover, the *Bacteroidetes*/*Firmicutes* ratio, as well as the *Bacteroidetes*-*Prevotella*/*C. coccoides*-*E. rectal* ratio, was positively correlated with plasma glucose concentration. Moreover, the class *Betaproteobacteria* was increased and positively correlated with reduced glucose tolerance [68,100]. Qin et al. and Karlsson et al. indicated that the gut microbiota of individuals suffering from T2D are characterized by increased amounts of opportunistic pathogens and decreased levels of *Faecalibacterium* and *Roseburia*, butyrate producers [69,70]. Qin et al. also reported an increased level of *Akkermansia muciniphila*, which shows mucin-degrading properties and plays an important role in gut barrier functions, and the sulphate-reducing species *Desulfovibrio* [69]. Moreover, Karlsson et al. showed an increased level of four *Lactobacillus* species and a decreased amount of five *Clostridium* species [70]. Zhang et al. indicated a decreased level of *Akkermansia muciniphila* in patients with T2D [71]. A similar observation was also made by Allin et al.—in individuals with prediabetes, there are fewer species of the genus *Clostridium* and *Akkermansia muciniphila*, in comparison to healthy people [72]. Conversely to Larsen et al., Sedighi et al. indicated a decreased level of *Bacteroidetes* and increased level of *Firmicutes* and *Proteobacteria*, which results in a higher *Firmicutes*/*Bacteroidetes* ratio [73]. These conclusions were confirmed later by Zhao et al., but they also elucidated that the enhanced *Firmicutes*/*Bacteroidetes* ratio was definitely higher in T2D-affected patients with complications of the disease than in individuals without complications [74].

The results of various studies differ from one another, but, in general, the genera negatively associated with T2D are *Bacteroides*, *Bifidobacterium*, *Faecalibacterium*, *Akkermansia* and *Roseburia*, and the genera *Fusobacteria*, *Ruminococcus* and *Blautia* are positively connected with this disease [101]. The inconsistent findings are the results of the inconsistency between studies. The DNA extraction protocols are not comparable between studies, nor are the sampling of specimens and the procedures using bioinformatic methods. Insufficient sample sizes and the interpersonal variation or environmental factors, such as the geographical locations, ages or gender, type of diet and medicaments, can be responsible for the discrepancies. Different methods, for instance microarrays, fluorescence in in situ hybridization or next-generation sequencing, lead to conflicting findings. Early studies were based on rRNA gene amplification by PCR, then on multiple sequence alignment and phylogenetic reconstruction. These studies were limited by the costs and time, which caused a problem in the exact estimation of the abundance of the microbiota corresponding to the sequences [102]. Moreover, research based on rodent microbiomes is more reliable when demonstrating probable mechanisms existing in human biology, rather than identifying exact taxa or species, because mouse microbiota are apparently different from human microbiota. Despite *Firmicutes* and *Bacteroidetes* being the dominant phyla, the composition at genus level is utterly different [103]. The protocols should be unified to enable definition of the exact changes of the gut microbiota in patients with T2D.

*Bacteroides*, belonging to the phylum *Bacteroidetes*, were negatively associated with T2D in the research [104,105,106]. Taking into consideration specific species, *B. intestinalis*, *Bacteroides* sp. *20_3* and *B. vulgatus* were decreased in T2D-affected patients [70,107,108]. In obese patients with T2D who had a laparoscopic sleeve gastrectomy and, subsequently, experienced diabetes remission, the level of *B. stercoris* was increased [109]. In animal studies, the administration of *B. acidifaciens* and *B. uniformis* favourably affected the glucose tolerance and IR in diabetic rodents [110,111]. Studies indicate a potentially beneficial effect of *Bacteroides* on glucose metabolism and suggest an explanation for the negative correlation between *Bacteroides* and T2D.

The genera *Roseburia*, *Faecalibacterium*, *Lactobacillus*, *Ruminococcus* and *Blautia* belong to the phylum *Firmicutes*. Generally, a decrease in the *Roseburia* level has been found in T2D-affected patients, in comparison to healthy individuals [68,104,108,112]. Considering specific species, *R. intestinalis* was positively and *R. inulinivorans* and *Roseburia_272* were negatively associated with diabetes [70,108,109]. *Faecalibacterium* were found to be decreased in T2D-affected patients, but, at species level, *F.* *prausnitzii* was negatively correlated with the disease [70,108,112,113]. *Lactobacillus* species are rather positively associated with T2D, for instance *L. acidophilus* or *L. salivarius*, but some species, such as *L. amylovorus*, are negatively associated with diabetes [70,104,107,114,115].

Species of the genus *Bifidobacterium*, belonging to *Actinobacteria*, are strongly negatively associated with T2D [73,104,107,113]. In animal studies, the administration of *Bifidobacterium* spp. Improved glucose tolerance in diabetic mice, suggesting a protective role of bifidobacterial in T2D [116,117].

A short comparison of these changes in the gut microbiota in individuals with diabetes is presented in Table 1.

## 6. The Potential Role of the Gut Microbiota in the Development of T2D

The development of T2D is mainly caused by insufficient insulin secretion by β-cells localized in the pancreas and the state called “insulin resistance”, which is the inability of insulin-sensitive tissues to respond to insulin properly [118]. The dysfunction of β-cells leads to a reduction in insulin secretion, resulting in accelerated glucose plasma levels. IR stimulates the production of glucose in the liver and impairs glucose uptake in the liver, muscle and adipose tissue, which increases glycemia as well. This situation leads to chronic hyperglycemia, affecting various organs and tissues, and resulting in detrimental micro- and macrovascular complications [119]. Chronic low-grade inflammation contributes to the development of IR and, consequently, of T2D [120]. Risk factors for T2D are genetic predisposition, ethnicity and family history of diabetes, as well as metabolic and environmental factors, such as obesity, low-grade physical activity and diet. The strongest risk factor is obesity, which is connected with metabolic changes leading to IR [121,122,123].

Much of our understanding of the exact role of the intestinal microbiota is based on studies focused on germ-free animals, which are born and kept without any contact with bacteria and can be exposed to specific microbes in the course of the research. Studies have shown that these rodents are resistant to obesity induced by diet [124,125], and exposure to *Enterobacter cloacae*, a bacterium linked to obesity, or bacteria received from obese donors leads to the increased capacity for energy harvest, weight gain and impaired glucose tolerance [126,127,128]. These studies suggest probable causality between the gut microbiota and obesity.

T2D is characterized by the decreased production of butyrate [97], one of the SCFAs that supports proper function of β-cells in the pancreas, especially after food intake [129]. Butyrate contributes to the modulation of immune system functions and protection against pathogen invasion [130]. It affects the functions of intestinal macrophages and downregulates pro-inflammatory mediators induced by LPSs, for instance IL-6, IL-12 and nitric oxide, as well as promoting regulatory T cell differentiation [131,132,133]. It also activates intestinal gluconeogenesis and, as a result, favorably affects glucose homeostasis [134]. Sanna et al. have shown that a host genetic-driven increase in the gut production of butyrate is associated with an improved insulin response following an oral glucose test, and that abnormalities in production or absorption of propionate are causally related to increased risk of T2D [129]. This state leads to low-grade inflammation [135,136]. In recent studies, it has been shown that dysbiosis occurring in NOD mice is associated with a reduction in butyrate levels, which leads to increased activity of histone deacetylase 3 (HDAC3), changed colon permeability, increased reactive oxygen species (ROS) production and a rise in IL-1β levels, as well as a decrease in amounts of IL-10 and IL-17α [137]. Moreover, it has been indicated that butyrate supplementation restores homeostatic levels of the inflammatory markers and reduces ROS production [137]. In an obese/prediabetic mouse model, butyrate intake has been proved to protect against the detrimental effects of high-fat diet, such as weight gain, body adiposity, IR, hyperglycemia and hyperinsulinemia [138].

Moreover, it is suggested that patients who will develop T2D in the future show a reduction in BCAA catabolism, as well as changes in lysophospholipid metabolism and in the BA pool [139]. Additionally, individuals with IR have an enhanced potential for biosynthesis of BCAAs [32]. As mentioned before, an increased BCAA level can be associated with a higher risk of developing IR [38]. Studies suggest that modifications of the BA pool by sequestrants can improve glycemic control in T2D-affected patients, but the mechanisms underlying these changes remain unknown [140].

**Figure 3:**
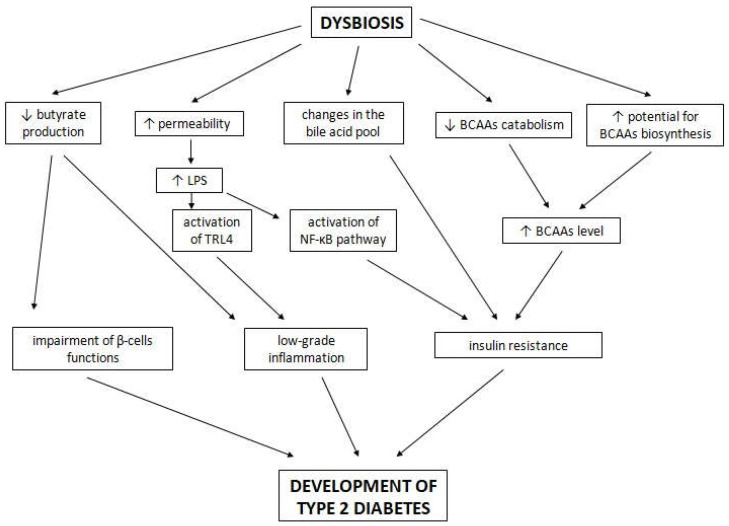
The possible influence of dysbiosis on type 2 diabetes development. A description is given in the text above.

Similar to T1D, T2D is probably associated with LPSs, which trigger the development of inflammation and IR acting through TLR4 [141,142]. TLR4 belongs to a family of pattern-recognition receptors, toll-like receptors, which contribute to the activation of pro-inflammatory signaling pathways, and cytokine expression and secretion in the presence of bacterial pathogens [143,144]. In vitro and in vivo studies have shown that free fatty acids can influence macrophages and adipocytes through TLR4, inducing inflammation. As a result, they are able to suppress insulin signaling through serine phosphorylation of insulin receptor substrate 1 (IRS-1) and influence glucose homeostasis [143,145]. This IRS-1 modification is considered to be a marker of the IR state [146]. It has been indicated that individuals with diabetes are characterized by higher fasting and postprandial concentrations of LPSs, in comparison to non-diabetic individuals, which can be caused by an increased permeability of the intestine and enhanced LPS absorption [141]. Increased circulating LPSs enhance the expression of inducible nitric oxide synthase (iNOS) through the activation of TLR4, which, as a consequence, induces protein S-nitrosation/S-nitrosylation of the insulin receptor, IRS-1 and Akt, and alters their proper functions [147,148,149,150,151]. Moreover, it has been suggested that metabolic endotoxemia dysregulates the inflammatory tone and triggers body weight gain and diabetes [141]. Probably, the gut microbiota have properties to modify this state of inflammation and endotoxemia due to their ability to affect the permeability of the intestine [152,153]. Endotoxemia in obesity and T2D becomes apparent, but there is still a lack of human studies that would show increased intestinal permeability and changed tight junction expression evidently. The possible influence of dysbiosis on T2D development is presented in Figure 3.

## 7. The Role of Roseburia Hominis, Faecalibacterium Prausnitzii and Akkermansia Muciniphila

The intestinal abundance of *Roseburia*_272 and *Faecalibacterium prausnitzii* is lower in T2D-affected individuals than in healthy ones [70,154]. The presence of the strictly anaerobic flagellated bacterium *Roseburia hominis*, belonging to the phylum *Firmicutes*, can contribute to the induction of genes involved in the promotion of gut barrier functioning and innate immunity, as well as in the promotion of mucosal T-cell expansion and differentiation of T cells [155]. Moreover, it is able to penetrate the mucous layer and adhere to the epithelial cells, which enhances its probiotic properties [156,157]. Hereby, *R. hominis* promotes and regulates the immune system.

*Faecalibacterium prausnitzii*, a representative of the phylum *Firmicutes*, has been shown to be one of the crucial producers of butyrate [158,159]. It is the most abundant bacterium in the intestinal microbiota occurring in healthy individuals [160]. It has proven anti-inflammatory properties, as it is able to induce a tolerogenic cytokine profile, decreasing acute, chronic and low-grade inflammation [161,162,163,164]. It is also one of the main butyrate producers [91,161]. Moreover, *F. prausnitzii* has an ability to produce salicylic acid, which has anti-inflammatory properties through the reduction of IL-8 levels [165]. It also synthesizes a protein called microbial anti-inflammatory molecule (MAM), which has been shown to have positive effects on gut inflammation and epithelial mucosa when supplemented in inflammatory bowel disease [166]. MAM probably regulates tight junction proteins and restores cell permeability, and thus influences the integrity of mucous intestinal cells [167]. It has been proved that the transplantation of *F. prausnitzii* can result in positive effects in the treatment of diabetes and its complications [168].

*Akkermansia muciniphila* is a bacterium belonging to the phylum *Verrucomicrobiota* and is responsible for mucin degradation in the gut lining, which, as a result, contributes to the syntropic interactions and stimulation of the metabolite pool in the intestine [169]. Moreover, it stimulates mucin synthesis, probably in an autocatalytic process [170,171]. In rodent studies, the colonization of the intestine by *A. muciniphila* has been shown to exert transcriptional changes manifesting as an increase in the expression of genes connected with immune processes and the metabolism of lipids [172,173].

## 8. Preventive and Therapeutic Perspectives including the Gut Microbiota

Diabetes worsens the quality of life of affected patients, leads to many early and late complications, burdens the health care system by increasing treatment costs and causes prolonged absence from work [174]; therefore, it is of great importance to create methods for alleviating the course of the disease. A promising approach is the modification of the amount and composition of the gut microbiota. A healthy diet and physical activity are among the factors that can influence the gut microbial ecosystem. In active women, in comparison to sedentary ones, an increased abundance of *Roseburia hominis*, *Akkermansia muciniphila* and *Faecalibacterium prausnitzii* [175] was observed. The Mediterranean diet and consumption of food substances, such as green tea, caffeine or omega-3 polyunsaturated fatty acids, help to restore the changed intestinal bacterial composition [176,177]. A carbohydrate-restricted or fat-restricted low-calorie diet used by obese patients has led to a renewed change in the *Firmicutes/Bacteroidetes* ratio [178,179]. Moreover, a fiber-rich diet is associated with increased amounts of *Prevotella*, while a protein-rich diet is related to an increased abundance of *Bacteroides* [180]. After 1 month of a strict vegetarian diet, 6 obese patients with T2D and/or hypertension had significantly reduced HbA_1c_ and triglyceride levels, decreased body weight and improved levels of fasting and postprandial glucose. Such a diet is associated with a reduced *Firmicutes*/*Bacteroidetes* ratio and increased amounts of *Clostridium* and *Bacteroides fragilis*, which lead to diminished intestinal inflammation and SCFA levels [181].

Prebiotics are fermentable, non-digestible food components that promote the growth of bacteria in the intestine [182]. The most popular prebiotics are inulin, lactulose, galactooligosaccharides and fructooligosaccharides, and they can alter the composition of the gut microbiota [182]. Two weeks of treatment with inulin-type fructans in healthy volunteers led to increased satiety, decreased postprandial glycemia and increased postprandial release of incretins [183]. According to a meta-analysis of 20 randomized controlled trials, the supplementation of inulin-type fructans correlates positively with decreased fasting insulin levels [184]. The arabinoxylans, a new class of prebiotics, are non-digestible carbohydrates appearing in wheat and that also have potential beneficial effects on glucose metabolism [185].

Probiotics are live microorganisms, either in the form of food or supplement, which can alter the gut microbiota [182]. *Lactobacillus* species are the major probiotics with a glucose-lowering potential [182]. In people without altered glucose tolerance, a daily intake of *Lactobacillus reuteri* enhances the secretion of insulin and incretin, but the effect can be bound with an improvement of β-cell function [186]. Moreover, in another study, it was indicated that in children carrying the high-risk HLA DR3/4, a genotype bound with T1D susceptibility, the risk of islet autoimmunity can be decreased by the early oral exposure to probiotics [187]. In patients with metabolic syndrome, levels of uric acid were significantly decreased and the total antioxidant capacity was significantly increased after consuming probiotic yoghurt containing *Lactobacillus acidophilus* La5 and *Bifidobacterium lactis* Bb12 for 8 weeks at a dose of 300 g/day [188].

Synbiotics are a combination of prebiotics and probiotics. Synbiotic supplementation has the potential to decrease the serum concentration of IL-6, TNF-α and hs-CRP, which are risk factors for inflammation-dependent cardiometabolic diseases, such as T2D [189]. In patients with T2D, a diet supplemented with either prebiotics or synbiotics has the potential to preserve glucose homeostasis and improve lipid metabolism [190]. Furthermore, the administration of symbiotics can cause a decrease in body weight and diminish anti-inflammatory activity [191].

Gluten intake affects the development of T1D via altering the gut microbial composition and the immune response [192]. This effect is modified by the amount, timing and mode of gluten intake [193,194,195]. A gluten-free diet can help to protect β-cell function by influencing the gut microbiota, which can be associated with the incidence of diabetes [192]. Furthermore, a high-fat diet can also change the composition of the intestinal microbiota, mainly by decreasing the amount of *Bifidobacterium* and. Administration of specific prebiotics can prevent the development of high-fat diet-induced diabetes and result in improved glucose tolerance, restored insulin secretion, decreased intestinal endotoxin levels and alleviated inflammatory response [196]. A deficiency of vitamin A increases the *Firmicutes*/*Bacteroidetes* ratio and decreases the level of bacteria producing butyrate [197]. Moreover, retinoic acid, a vitamin A metabolite, can inhibit the differentiation of pro-inflammatory Th17 cells and promote the differentiation of anti-inflammatory Treg cells [198]. These mechanisms protect against the development of T1D. Furthermore, zinc deficiency influences the inflammatory response and metabolic control, which can promote T1D incidence [199].

Another therapeutic strategy is fecal transplant. Six weeks after the infusion of the gut microbiota from lean donors to male recipients with metabolic syndrome, the levels of butyrate-producing intestinal microbiota and the insulin sensitivity of the recipients were significantly increased [168]. De Groot at al. showed that, in patients recently diagnosed with T1D, fecal microbiota transplantation in the 12 months after disease onset can halt the decline in endogenous insulin production, probably by the preservation of residual β-cell function [200]. Moreover, 3 weeks after the oral transfer of fecal bacteria in another study, the abundance of *Lachnospiraceae* and *Clostridiaceae* was increased and the amount of *Lactobacillaceae* was decreased, which indicates a possible improvement in insulin sensitivity in diabetic patients [201].

## 9. Lacking Data and Future Directions

Although much research about diabetes and the gut microbiota has already been performed, we are still at the beginning of the way to show the exact role of the intestinal microbiota in T1D or T2D. Until now, we have frequently based our theories on studies with rodents, but mouse microbiota differ significantly from those in humans. Moreover, the germ-free animals used in experiments are born and kept without any contact with bacteria, and are exposed to selected microbes during the research process. Retrospective and observational research has been performed that does not exactly allow the analysis of the causal relationship between gut microbiota and diabetes development. We still do not know whether the intestinal microbiota are solely involved in β-cell autoimmunity or can also activate T1D. Verification of this hypothesis will require the performance of longitudinal, interventional and prospective studies with adequate methodology and the use of human stool sample processing. Additionally, standardized and reproducible methods of analyzing genetic material are needed. Furthermore, we do not possess enough evidence to definitively prove that dysbiosis can cause T2D, or whether it just appears during the course of diabetes as a consequence of metabolic changes connected with the disease. Further efforts should be directed to creating such studies, which will allow the demonstration of a causal relationship between the changes in intestinal microbiota and diabetes.

## 10. Conclusions

It is of great importance to understand the exact mechanisms underlying the diseases of civilization, as their prevalence is still rising. Diabetes is one of the most common metabolic disorders and leads to serious complications and consequences. The gut microbiota is an inseparable part of human beings, and understanding its exact role in the functioning of living organisms is necessary. It is of great importance to prove whether there exists the causal relationship between diabetes development and the gut bacteria. Further research is needed, particularly unified studies that can clearly indicate exactly how the microbiota change and how they influence the host, to make the most of the potential included in the gut microbiota.

## Figures and Tables

**Table 1:** A comparison of changes in the gut microbiota occurring in individuals with type 1 diabetes or type 2 diabetes in comparison to healthy individuals. A description is given in the text above. ↑—increased level, ↓—decreased level.

Individuals with Type 1 Diabetes in Comparison to Healthy Ones	Individuals with Type 2 Diabetes in Comparison to Healthy Ones
Giongo et al. (2011) [61] ↑ *Firmicutes*/*Bacteroidetes* ratio and instability of microbiota are early diagnostic markers of developing autoimmune disorders, such as T1D De Goffau et al. (2014) [62] ↑ *Bacteroidetes* ↑ *Streptococcus mitis* ↓ *Lactobacillus plantarum* ↓ *Clostridium* clusters *IV* and *XIVa* Mejía-León et al. (2014) [63] ↑ *Bacteroides* ↓ *Prevotella* After 2 years of insulin treatment, no significant differences Vatanen et al. (2018) [64] ↑ *Roseburia hominis* ↑ *Alistipes shahii* ↑ *Bifidobacterium pseudocatenulatum* ↓ *Lactococcus lactis* ↓ *Streptococcus thermophilus* ↓ *Lactobacillus rhamnosus* ↓ *Bifidobacterium dentium* ↓ Genes associated with SCFA production and fermentation Pellegrini et al. (2017) [65] Characteristic inflammatory profile ↑ *Firmicutes* ↑ *Firmicutes*/*Bacteroidetes* ratio ↓ *Proteobacteria* ↓ *Bacteroidetes* Siljander et al. (2019) [66] ↑ *Bacteroidetes* ↓ *Firmicutes*	Larsen et al. (2010) [68] ↑ *Bacteroidetes* ↑ *Bacteroidetes*/*Firmicutes* ratio and *Bacteroidetes*-*Prevotella*/ *C. coccoides*-*E. rectal* ratio ↑ Class *Betaproteobacteria* ↓ Phylum *Firmicutes* ↓ Class *Clostridia* Qin et al. (2012) [69] ↑ Opportunistic pathogens ↑ *Akkermansia muciniphila* ↑ *Desulfovibrio* ↓ *Faecalibacterium* ↓ *Roseburia* Karlsson et al. (2013) [70] ↑ Four *Lactobacillus* species ↓ Five *Clostridium* species ↓ *Roseburia intestinalis* ↓ *Faecalibacterium prausnitzii* Zhang et al. (2013) [71] ↓ *Akkermansia muciniphila* Allin et al. (2018) [72] ↓ Genus *Clostridium* ↓ *Akkermansia muciniphila* Sedighi et al. (2017) [73] and Zhao et al. (2019) [74] ↑ *Firmicutes* ↑ *Proteobacteria* ↑ *Firmicutes*/*Bacteroidetes* ratio ↓ *Bacteroidetes*

## Data Availability

Not applicable.
